# Novel Prognostic Immunohistochemical Markers in Uveal Melanoma-Literature Review

**DOI:** 10.3390/cancers13164031

**Published:** 2021-08-10

**Authors:** Malgorzata Gajdzis, Radoslaw Kaczmarek, Pawel Gajdzis

**Affiliations:** 1 Department of Ophthalmology, Wroclaw Medical University, 50-556 Wroclaw, Poland; gosiagajdzis@gmail.com; 2 Department of Clinical and Experimental Pathology, Wroclaw Medical University, 50-556 Wroclaw, Poland; pawel.gajdzis@umed.wroc.pl

**Keywords:** uveal melanoma, prognostic factors, proteins, immunohistochemistry

## Abstract

**Simple Summary:**

The following study provides an overview of the English-language literature on the search for new prognostic factors for uveal melanoma. Uveal melanoma is the most common primary intraocular tumor in adults, and although it is a relatively rare disease, it poses a significant health problem. About half of the patients develop distant metastases, and unfortunately there are currently no effective treatments for the disease at such an advanced stage. The search for new prognostic factors is important to understand the biology of the disease and to be able to monitor patients more effectively. At the same time, it creates an opportunity to find new therapeutic targets. We focused our attention on immunohistochemical research because it is a widely used method, relatively cheap, technically simple, and gives reproducible results. The analysis of this study will enable other researchers to verify their research plans and may also be a source of inspiration for creating new ones.

**Abstract:**

Uveal melanoma is the most common primary intraocular neoplasm in adults. As there are currently no effective methods of treating the disease in the metastatic stage, there is a need to search for new prognostic factors that would enable a reliable assessment of the patient’s condition and constitute a possible therapeutic target. In this review, we have prepared the results of English-language studies on new prognostic factors determined with immunohistochemical methods. We found 64 articles in which the expression of various proteins was associated in a statistically significant manner with the histopathological and clinical prognostic factors identified by AJCC. The results of our work clearly show that the biology of uveal melanoma is extraordinarily complex. Numerous studies have shed new light on the complexity of the processes involved in the development of this cancer. Moreover, a careful analysis of the expression of individual proteins may allow the identification of homogeneous groups of patients requiring different treatment regimens.

## 1. Introduction

Uveal melanoma (UM) is the most common primary intraocular malignancy in adults and constitutes a significant problem in ophthalmic oncology. The incidence remains stable over many years of follow-up, at approximately 5.1 cases per million per year [1]. The most important risk factors are ethnicity, age, fair skin, light eye color, tendency to sunburn, ocular melanocytosis, iris or choroidal nevus, and BRCA1-associated protein 1 mutation [1].

The uvea forms the middle layer of the eye, bordering on the inside with the retina and on the outside with the sclera. It consists of the iris, the ciliary body, and the choroid [2]. The uvea is a richly perfused structure consisting of blood vessels and the stroma surrounding them. Within the stroma there are melanocytes from the neural crest, from which melanomas arise [3]. Choroidal melanomas account for 85–90% of all UM. Much less often the disease develops in the ciliary body (5–8%) and the iris (3–5%) [4].

There is no basement membrane within the uvea, so UM is in direct contact with the blood vessels, making it much easier for the disease to spread [2]. Metastases to the regional lymph nodes (parotid, submandibular, or cervical) appear late and only when the outer parts of the sclera and conjunctiva are affected. The vast majority of distant metastases occur in the liver (60–90%), followed by the lungs, subcutaneous tissue, bones, and brain [5]. The COMS study showed that 93% of patients have liver metastases at death, and in cases with only one site of metastasis, the liver is involved in 95% [6].

Five-year survival rates in UM range from 25% to 97% (mean 69%), depending on the clinical stage of the disease (staging) [7]. The prognosis worsens significantly in the presence of distant metastases—then the survival rates are as low as 8% after two years [5]. The average survival time from detection of the presence of metastases is 4–15 months [8]. Over the last thirty years of observation, the survival times have not changed significantly. Many treatments modalities have been evaluated for effectiveness in metastatic disease; unfortunately, none of them have resulted in an increase in survival [9]. A meta-analysis of 29 studies conducted between 1988 and 2015 showed that the average progression-free survival and overall survival have not changed over the years, regardless of the therapies used [3].

Currently, the treatment of UM is focused on the primary tumor. Radiotherapy is the most commonly used; less often, tumor resection, or in the case of advanced disease, removal of the eyeball (enucleation) [3]. In small lesions, transpupillary thermotherapy or photocoagulation can be used, but their usefulness is limited due to numerous side effects [10]. There are also attempts to administer drugs by injection into the vitreous chamber [3]. Topical treatment is highly effective. Unfortunately, half of the patients develop metastases, often many years after the primary tumor has been treated [11]. This inevitably leads to death as there is no effective treatment for the systemic disease.

The search for new reliable prognostic factors is important as it allows the identification of groups of patients more prone to metastasis. It acquires additional significance in the context of the lack of clear guidelines regarding the intervals between control tests and the type of tests that should be performed during the control. Assigning patients to appropriate groups allows for more frequent monitoring of patients from higher risk groups. Detecting distant metastases at an early stage makes it possible to remove them surgically, which is currently the only proven way to extend patients’ lives [12]. Therefore, it seems justified to search for new markers, especially in the context of a holistic approach to the patient and adjusting the therapy to individual needs. Moreover, potential prognostic factors may also become a target of therapy.

In recent years, significant progress has been made in the development of molecular methods that make it possible to predict the course of the disease based on the analysis of genetic material. Initially, based on the gene expression profile, UM were divided into two groups—class 1 associated with low risk of metastasis, class 2 with high. However, it quickly turned out that a large proportion of patients classified as class 1 died because of the development of distant metastases. For this reason, class 1 was divided into two subclasses—1A and 1B, based on the expression of the PRAME marker [13]. In class 1A, the risk of developing metastases within 5 years is 2%, while in class 1B it is 21%. A recently published report, part of the Cancer Genome Atlas project, categorized UM into four groups [14]. The results of the report indicate a high prognostic value of genetic abnormalities in UM, but no prospective studies to support the results are yet available. Proponents of molecular methods are of the opinion that genetic testing is sufficient to determine the prognosis and identify groups of patients at increased risk. However, it has been shown that the most accurate prediction can be obtained by combining the TNM classification with genetic tests, in particular the loss of chromosome 3 [15].

The AJCC tumor staging is a recognized, long-established system, thoroughly tested and regularly updated. The latest, 8th edition, describes the basic, recognized prognostic factors on which the TNM classification is based, as well as other, relatively new, independent prognostic factors, including the gene expression profile [7]. AJCC members emphasize that genetic testing, while highly promising, cannot be the primary and only prognostic factor. The main weaknesses are the short follow-up time of patients (on average less than 5 years) and the small number of patients in many T-categories. It should also be remembered that the AJCC anatomical classification will remain the gold standard for patients where genetic testing will not be available.

The AJCC classification takes into account the prognostic factors necessary for the stage grouping (TNM) and additional factors recommended for clinical care—size, location, extraocular extension, cell type, chromosomal analysis, gene expression profiling, mitotic count, extravascular matrix loops and networks, microvascular density, and tumor-infiltrating macrophages and lymphocytes. For the purposes of this study, we have researched the English-language literature available in the PubMed medical database. We searched for articles on protein expression determined by immunohistochemistry (IHC), with statistically significant relationships with the AJCC-listed prognostic factors for uveal melanoma. We decided to focus on IHC, because it is a technique that is widespread all over the world, gives highly reproducible results, and is relatively cost-effective [16]. This makes it one of the most universal tools in cancer diagnosis and prognosis. We focused on new, promising proteins that could also be an important therapeutic target in the future. A summary table in alphabetical order can be found at the end of the article (Table 1).

## 2. Tumor Size

Largest basal tumor diameter is the predominant clinical predictor of prognosis, strongly associated with risk of metastasis (AJCC Level of Evidence: I) [7]. Studies also show that tumor thickness may be an independent prognostic factor [72]. The most accurate measurements are obtained by analyzing fundus photography and ultrasonography. In the event of enucleation, the measurement may be understated due to tissue shrinkage during material processing.

Ataxia-telangiectasia mutated protein (ATM) is an activator of DNA damage response. It is predominantly nuclear protein. Loss of expression correlates with both large tumor diameter and tumor thickness [21]. With tumor thickness correlate high expression of c-REL and CCR7 [26,35]. c-REL is a member of the nuclear factor κB (NF-κB) transcription factor family and an emerging regulator of tumorigenesis. CCR7 (C-C Motif Chemokine Receptor 7) is a receptor mainly expressed in lymphoid cells. It mediates cell migration of naïve lymphocytes and mature dendritic cells to secondary lymphoid organs and regulates the transport of cancer cells through the extracellular matrix.

In the literature, we found several proteins for which the relationship of expression with the largest tumor diameter has been demonstrated. nm23, a product of the metastasis suppressor gene (NM23), is expressed in cytoplasm. Low expression correlates with the largest tumor diameter [57]. In other cases, tumor diameter is related to high protein expression. They are Hsp90, PARP, and PD-1 [46,60,63]. Heat shock protein 90 (Hsp90) plays a role in folding, intracellular transport, maintenance, and degradation of proteins, and facilitates cell signaling. Poly (ADP-ribose) polymerase (PARP), a nuclear protein, participates in the DNA repair processes. Programmed cell death receptor-1 (PD-1) is a membrane-bound receptor and plays a role in regulation immune system’s response to the cells of the human body.

High expression of cluster of differentiation 147 (CD147), a member of the immunoglobulin superfamily, which plays a role in intercellular recognition, various immunologic phenomena, differentiation, and development, also correlates with larger tumor diameter, but only in the nonmetastatic sub-group [28].

Another relationship occurs in the case of EphA1, the erythropoietin-producing human hepatocellular receptor A1, where high expression is associated with a smaller tumor diameter [43]. Thus, high expression is a favorable prognostic factor in this case. EphA1 is an RTK receptor and plays a role in the regulation of a processes critical to embryonic development, including axon guidance, formation of tissue boundaries, cell migration, segmentation, proliferation, and angiogenesis.

Proteins whose expression is related to tumor size are listed in Table 2.

## 3. Tumor Location

Involvement of the ciliary body in the disease process is associated with the least favorable prognosis. It is independently associated with the risk of metastasis (AJCC Level of Evidence: I) [72]. Due to its location, the ciliary body is poorly accessible in the examination. For this reason, possible neoplastic changes may develop long before they are detected. It is nearly impossible to visualize the ciliary body directly. Its condition can be most accurately assessed in anterior segment ultrasound imaging. Other options available are gonioscopy, transillumination, and optical coherence tomography of the anterior segment of the eye [7].

In the literature, we found four proteins whose high expression is associated with the involvement of the ciliary body. These include the previously mentioned PD-1 and CD147, but in the case of CD147, the relationship relates only to the metastatic sub-group [28,62]. The other two are HERC2 (HECT and RLD Domain-Containing E3 Ubiquitin Protein Ligase 2), a predominantly nuclear and cytoplasm protein, which plays a role in DNA repair regulation, pigmentation, and neurological disorders, and P-protein (pink-eyed dilution protein), which plays a role in melanin synthesis in melanocytes and retinal pigment epithelium [45].

Proteins whose expression is related to ciliary body involvement are listed in Table 3.

## 4. Extraocular Extension

The prevalence of extrascleral infiltration is an unfavorable prognostic factor (AJCC Level of Evidence: II). If it occurs at the front part of the eyeball, it can be assessed by a basic ophthalmological examination. Posterior infiltration can be assessed by imaging studies—ultrasound, computed tomography, or magnetic resonance imaging [7]. In the case of removal of the eyeball, histopathological examination can assess scleral invasion, which is an event immediately preceding the formation of extrascleral infiltration [72].

Low expression of the above-mentioned nm23 is associated with the presence of a deeper scleral invasion [57]. On the other hand, in the case of BNIP3 (BCL2 19 kD protein-interacting protein 3), protein, which regulates cell death, autophagy, and cytoprotection, high expression in the cytoplasm is associated with a deeper scleral invasion [24].

High EphA1 expression is a favorable prognostic factor as it is associated with a lower incidence of exstrascleral infiltration [43]. In contrast, in the case of Cripto-1 (teratocarcinoma-derived growth factor-1) and Cyclin1, high expression increases the risk of the presence of extrascleral infiltration. Cripto-1 is an oncogenic growth factor involving tumorigenesis and cancer cell proliferation and survival [36]. Cyclin1 is a predominantly nuclear protein and regulator of the cell cycle. Its relationship with the formation of extrascleral infiltrates has been demonstrated in two studies [38,39]. Finally, low expression of adiponectin correlates with extrascleral infiltration. Adiponectin is a protein hormone involved in regulating glucose levels, fatty acid breakdown, and plays a role in limiting cell proliferation and reducing inflammation [19].

Proteins whose expression is related to scleral and extrascleral infiltration are listed in Table 4.

## 5. Cell Type

The type of cells present in the tumor tissue is an independent prognostic factor associated with the risk of metastasis (AJCC Level of Evidence: I) [7]. There are two types of cells in UM—spindle and epithelioid cells. Both types of cells can appear within the tumor, and the presence of epithelioid cells is associated with a worse prognosis. There is no consensus on the proportions of individual cells required to qualify a given tumor as mixed or epithelioid type. Many pathologists do not try to classify individual types, and instead only determine the presence of epithelioid cells [7].

The presence of epithelioid cells is associated with high expression of the aforementioned c-REL, CyclinD1, HERC2, PD-1, and P-protein [35,39,45,62]. In the case of PARP, high expression correlates with a higher histopathological grade [60]. In turn, low expression of ATM correlate with the presence of epithelioid cells [21]. Low expression of p16 (cyclin-dependent kinase inhibitor 2A, CDKN2A, multiple tumor suppressor 1), an inhibitor of cyclin-dependent kinases responsible for slowing the progression of the cell cycle from G1 phase to the S phase, is associated with mixed cell type [59].

With the presence of epithelioid cells, high expression of LOX (lysyl oxidase), an extracellular enzyme, also correlates, playing a role in embryonic development, wound healing, and adult tissue remodeling [50]. Nestin (neural stem cell protein), a cytoplasmic and membrane-bound protein, a member of the intermediate filament (IF) class VI protein family, correlates with the presence of epithelioid cells too [56]. Moreover, high CEACAM expression (carcinoembryonic antigen cell adhesion molecule-1), a transmembrane glycoprotein, playing a role in the intercellular interactions, regulation of cell growth, angiogenesis, immune modulation, and hepatic insulin clearance, is associated with the presence of epithelioid cells, as is the case with CXCR4 (C-X-C motif chemokine receptor 4), an alpha-chemokine receptor specific for stromal-derived-factor-1, a molecule endowed with potent chemotactic activity for lymphocytes [29,37].

In contrast, in the case of NEMO/IKKγ (nuclear factor κB essential modulator, inhibitor of nuclear factor kappa B kinase subunit gamma), a protein essential for the activation of transcription factor NFκB, which regulates the cellular responses to inflammation, immunity and cell survival, low expression correlates with presence of epithelioid cells [55].

Proteins whose expression is related to presence of various cell type are listed in Table 5.

## 6. Chromosomal Analysis

Another independent prognostic factor associated with the risk of metastasis is the loss of chromosome 3, especially with the frequently coexisting gain in chromosome 8q (AJCC Level of Evidence: II). The combination of both, monosomy 3 and chromosome 8q gain, are associated with the worst prognosis. Chromosome status is typically determined by karyotyping or fluorescent in situ hybridization [7].

We found only five studies in the literature in which the expression of specific proteins was related to the status of chromosomes. High EphA5 expression seems to be a favorable prognostic factor as it correlates with less frequent loss of chromosome 3 [43]. In turn, high PARP expression is associated with more frequent loss of chromosome 3 [60]. The worst prognosis induces high nestin expression, as it is associated with more frequent loss of chromosome 3 and chromosome 8q gain [56].

The next study we found is about PERP protein (p53 apoptosis effector related to PMP-22), playing a role in inducing cell death. In his case, low expression is associated with more frequent loss of chromosome 3 [64]. An analogous situation occurs in the case of adiponectin, where low expression also correlates with more frequent loss of chromosome 3 [19].

Proteins whose expression is related to chromosome status are listed in Table 6.

## 7. Mitotic Count

Mitotic count is independently associated with metastatic risk (AJCC Level of Evidence: II) [7]. Higher counts are associated with shorter survival. Mitotic count is assessed with a light microscope, counting at least 40 high-power fields [73]. Alternatively, mitotic cells can be assessed using immunohistochemical methods.

High expression of nestin correlates with higher mitotic count [56]. The same relationship occurs in the case of EGFR (epidermal growth factor receptor), a transmembrane protein that plays a role in epithelial tissue development and homeostasis [40]. High expression of EphA1 correlates with a lower mitotic count, while low expression of NEMO/IKKγ correlates with a higher mitotic count [43,55].

Proteins whose expression is related to mitotic count are listed in Table 7.

## 8. Extravascular Matrix Loops and Networks

Two types of extravascular matrix pattern have been documented that are associated with a higher risk of metastasis (AJCC Level from Evidence: II) [13]. These are networks and loops formed by the tumor vessels. The presence of patterns is determined under a light microscope [7].

High CEACAM expression is associated with the presence of the networks [29]. In turn, high expression of MMP-9 and VEGF-A is associated with the presence of both loops and networks [52]. MMP-9 (matrix metalloproteinase-9) is secreted along with membrane-associated neutral endopeptidase, and plays a role in degrading extracellular matrix proteins, cell proliferation, migration, differentiation, angiogenesis, apoptosis, and host defense. VEGF-A (vascular endothelial growth factor-A) acts specifically on endothelial cells, mediates increased vascular permeability, induces angiogenesis, vasculogenesis, and endothelial cell growth, promotes cell migration, and inhibits apoptosis.

Low expression of NEMO/IKKγ and high expression of nestin correlates with the presence of vascular loops [55,56].

Proteins whose expression is related to extravascular matrix loops and networks are listed in Table 8.

## 9. Tumor-Infiltrating Macrophages and Lymphocytes

Tumor-infiltrating macrophages (TIMs) and tumor-infiltrating lymphocytes (TILs) are other important prognostic factors in uveal melanoma. Generally, an inflammatory infiltrate is associated with a worse prognosis [13]. The AJCC lists the presence of TIMs as being independently associated with the risk of metastasis (AJCC level of Evidence: II) [7].

High expression of the three proteins is associated with a more intense infiltration of TIMs. They are PD-1, PD-L1, and EMAP-II [42,62]. EMAP-II (endothelial monocyte-activating polypeptide II) is a proinflammatory cytokine and chemoattractant of macrophages, expressed on the cell surface. In the case of TILs, a relationship was also demonstrated between the intensity of infiltration and high expression of the three proteins. They are CCR7, MMP-9, and VEGF-A [26,52].

Proteins whose expression is related to TIMs, and TILs infiltration are listed in Table 9.

## 10. Other Prognostic Factors

In most studies on prognostic factors, the authors try to demonstrate the relationship of the studied proteins with as many parameters as possible. Some of them do not currently have a confirmed prognostic value, but they are certainly worth paying attention to, as they constitute a significant contribution to expanding knowledge about the biology of UM. In this section, we list proteins whose expression is associated with these parameters. We also included some research related to the AJCC staging group.

High expression of BNIP3, HERC2, and P-protein is associated with increased pigmentation within the tumor [24,45]. The amount of the pigment probably does not influence the prognosis in melanoma in any way, but it has some diagnostic significance as it hinders the immunohistochemical assessment of the examined tissues. High P-protein expression is also associated with more advanced clinical tumor staging [45]. A similar relationship occurs in the case of PD-L1 and CD147, with the latter only in the nonmetastatic subgroup [28,62]. The inverse relationship is observed in the case of PLK-1 (polo-like kinase-1), a regulator of mitotic entry and cytokines. Its low expression correlates with the higher AJCC prognostic stage group [66].

Another parameter is the severity of necrosis in the tumor tissues. Necrosis usually occurs in advanced tumors and is therefore associated with poor prognosis [74]. In UM, the severity of necrosis was associated with high CCR7, MMP-9, and VEGF-A expression [26,52].

High expression of EphA1 and EphA5 is associated with more frequent hemorrhages in the vitreous chamber in patients with UM [43]. This dependence probably results from the role played by Eph receptors in the angiogenesis process. In turn, low NEMO/IKKγ expression is associated with the presence of neovascularization in the tumor [55]. The latest relationships we found are high expression CyclinD1 related to the tumor cell MIB-1 positivity and high PD-1 expression related to the absence of BAP-1 staining [39,62].

Table 10 summarizes the relationships discussed above.

## 11. Metastasis

As mentioned earlier, the development of metastases significantly worsens the prognosis for patients with UM. Because metastases can appear up to 30 years after primary tumor treatment, patients must be closely monitored for the rest of their lives [7]. There are no strict guidelines for the management of patients after UM is detected. As agreed by COMS, a physical examination and liver function test are not sufficient to detect possible metastases. Usually, imaging tests (ultrasound, MR imaging, or CT) are performed annually, but the procedures may differ in individual centers [75]. Since only a quick detection of metastases in the liver followed by their surgical removal may slightly extend the survival time of patients, it is important to search for new prognostic factors that would allow to select groups of patients at higher risk. Therefore, most of the studied proteins are tested for dependence on the occurrence of metastasis.

In most of the studied cases, high expression of a given protein is associated with more frequent metastases. Of the proteins mentioned earlier, this is the case with CCR7, c-REL, Cripto-1, CyclinD1, MMP-2, MMP-9, PD-1, PD-L1, P-protein, and VEGF-A [27,28,35,36,39,45,52,62]. High expression of many consecutive proteins is also associated with more frequent occurrence of metastases. ABCB5 (ATP-binding cassette sub-family B member 5), human transmembrane P-glycoprotein, plays a role in transmembrane transport (including chemotherapeutic drugs), which makes it particularly important in the context of drug resistance [17]. ADAM10 (a disintegrin and metalloproteinase domain-containing protein 10), a transmembrane protein, controls membrane fusion and cell-cell and cell-matrix interactions [18]. c-Met (Tyrosine-protein kinase Met or hepatocyte growth factor receptor (HGFR)), a transmembrane RTK receptor, plays a role in embryonic development, organogenesis and wound healing, angiogenesis, and metastasis formation [30]. C-NFκB proteins (canonical nuclear factor-κB proteins (p65 and p50)), coordinate innate immunity and inflammation processes, particularly important in neoplastic transformation [32]. Other proteins associated with the presence of metastasis are COX-2 (Cyclooxygenase-2), an enzyme which catalyzes the prostanoid synthesis reaction [34], and p53, a cell-cycle regulatory protein [39]. PCNA (proliferating cell nuclear antigen, ATLD2), the DNA polymerase auxiliary protein involved in the control of DNA replication, is also associated with the presence of metastases [61]. Similar relationships are observed in the case of phospho-Akt, a cytoplasmic protein that plays a role in phosphorylation and inactivation of several proteins involved in apoptosis [65], and PRDX3 (thioredoxin-dependent peroxidase reductase), a cytoplasmic protein involved in redox regulation of the cell and protects radical-sensitive enzymes from oxidative damage [67]. Finally, SPANX-C (SPANX family member C; Sperm protein associated with the nucleus on the X chromosome C), a cytoplasmic protein, is expressed in highly metastatic cell lines [69].

For a few proteins, high expression is associated with a higher likelihood of death from metastasis. These are the previously described EGFR and COX-2, and IGF-1R and MCAM [33,41]. IGF-1R (insulin-like growth factor 1 receptor), a transmembrane receptor implicated in insulin signaling, plays a role in several cancer development [48]. MCAM (melanoma cell adhesion molecule, MUC18, Mel-Cam, CD146), an adhesion molecule, plays a role in intracellular signaling cascades [51]. In turn, high expression of LOX and NC-NFκB proteins is associated with reduced metastasis-free survival time [50,54]. NC-NFκB proteins (p52, RelB, and co-expression of p52/RelB) are nuclear proteins; they play a role in promoting cancer proliferation and progression. In the case of syntenin (syndecan binding protein syntenin-1, melanoma differentiation-associated gene 9, mda-9), a predominantly cytoplasm protein playing a role in clustering of membrane receptors, intracellular trafficking, Sox4 activation, and signal transduction, high expression correlates with the risk of recurrence of metastasis [71].

Low adiponectin expression is associated with the presence of metastases [19]. A similar relationship occurs in the case of RKIP (Raf kinase inhibitor protein), a regulator of proliferative pathways within the cell [68]. The presence of metastases is also associated with the loss of ICAM expression (intercellular cell adhesion molecule-1), an adhesion molecule and ligand for leukocyte function-associated antigen-1, involved in the process of inflammation, the circulation of blood cells, and in the immune surveillance of the host [47].

High expression of the aforementioned EphA5 is associated with less frequent occurrence of metastases [43]. A similar situation occurs in the case of beclin, an autophagy related protein, performing a central role in the autophagic process as a major member of the macro-autophagic phase [23].

Table 11 summarizes the relationships discussed above in alphabetical order.

## 12. Survival Times

From the point of view of oncology, establishing survival times is of fundamental importance. It is an extremely clear picture of the prognosis for the patient. For this reason, many studies have focused on linking the expression of various proteins with survival times, even though such studies require a long time of observation.

In UM, most of the proteins tested show a reduction in survival times with high expression. Such relationships have been demonstrated for BNIP3, CCR7, c-Met, C-NFκB proteins, c-REL, MMP-2, and MMP-9, Nestin, PARP, PD-1, and PRDX3 [24,26,28,30,32,35,56,60,63,67]. Proteins whose high expression also is associated with a reduction in survival times are also AIF, JARID1B, legumain, Nbs1, and NC-NFκB proteins.

AIF (apoptosis inducing factor) is ubiquitous protein and plays a proapoptotic function in the nucleus and redox activity in mitochondria [20]. JARID1B (Jumonji AT-rich interactive domain 1B), a demethylase enzyme, induces demethylation of tri- and di-methylated lysines in the 4 position of histone 3 [49]. Legumain (asparagine endopeptidase (AEP)), a proteolytic enzyme, plays a role in the functioning of the immune system [76]. Nbs1 (Nibrin, NBN), an intracellular protein, plays a role in the repair of double strand breaks and telomere maintenance [53]. NC-NFκB proteins (p52, RelB, and co-expression of p52/RelB), which are nuclear proteins, plays a role in promoting cancer proliferation and progression [54].

At the opposite site, there are relationships where high expression in immunohistochemical studies is associated with longer survival. Among the previously mentioned proteins, this is the case of Beclin and EphA5 [23,43]. Other factors are BTNL9, HER3, nm23-H1, SSR, TIMP-1, and TIMP-2.

BTNL9 (butyrophilin-like protein 9) is a modulator of the T cell response [25]. HER3 (human epidermal growth factor receptor 3 or receptor tyrosine-protein kinase erbB-3), a transmembrane RTK receptor, is implicated in growth, proliferation, chemotherapeutic resistance, and the promotion of invasion and metastasis [44]. nm23-H1 is a cytoplasm protein, the product of a metastasis suppressor gene (NM23) [58]. SSR (somatostatin receptor) through the binding of somatostatin starts a signaling pathway that leads to arrest of cell growth or apoptosis [70]. TIMP-1 and TIMP-2 (tissue inhibitor of metalloproteinase-1 and -2) are metalloproteinase inhibitors [28].

Moreover, low or loss of expression may be associated with a reduction in survival time. Such relationships have been demonstrated for ATM, NEMO/IKKγ, and PLK-1 [18,39,45]. In the literature, we also found articles on the relationship of high c-Met and IGF-1R expression to melanoma-specific mortality [30,40].

Table 12 summarizes the relationships discussed above in alphabetical order.

## 13. Therapeutic Perspectives

The described protein markers of UM are not only a diagnostic tool but may also be a potential target in anti-cancer therapy, especially in the case of advanced UM. As there is currently no effective pharmacological treatment for such patients, they may be crucial for favorable long-term survival. Some of them have already been tried to be used for therapy, and others are still waiting for their chance. One of the better described in this regard are immune checkpoint inhibitors (ICIs). Their introduction started a new era in the treatment of cancer. ICIs restore the immune system’s ability to perform a standard, cytotoxic response against cancer cells. Antibodies targeting CTLA-4 and PD-1 in the treatment of cutaneous melanoma (CM) significantly improved treatment outcomes in metastatic CM. Unfortunately, these results were not confirmed in clinical trials of UM patients. Studies with ipilimumab, tremelimumab, nivolumab, and pembrolizumab were somewhat disappointing [77,78,79,80,81,82,83]. Slightly better results were achieved in combination therapy in which drugs against both types of ICIs receptors were used: CTLA-4 and PD-1, but still without a satisfactory breakthrough [84,85].

Another group of biomarkers targeted for cancer therapy is RTKs: IGFR, EGFR VEGFR, c-Met, and its ligands. In recent years, numerous studies have been published on the effectiveness of their use in treating metastatic UM, with both monoclonal antibodies or small molecule drugs targeting the extracellular domains of RTKs or RTK ligands. These studies, however, often concerned unselected, small groups of patients. For example, in the case of IGFR, a phase I trial with a monoclonal antibody against IGF-1R in patients with relapsed multiple myelomas did not provide satisfactory results [86]. However, more recent in vitro reports of ICF-1R blocking with pristimerin are more promising [87].

In the EGFR inhibitor, gefitinib, a decrease in UM cells survival was found in in vivo studies [88]. IGFR inhibition with gefitinib in the phase II study showed only low clinical efficacy in the group of unselected patients [89]. Intraocular VEGF inhibitors have revolutionized the treatment of the wet form of AMD, but their pedigree comes from oncological therapy, where they have so far been found in the treatment of numerous solid tumors. They were also tested for use in UM. However, a retrospective analysis of metastatic UMs treated with one of the anti-VEGF antibodies, bevacizumab, compared to the group not receiving this antibody, showed no statistical difference [90]. The other axitinib and pazopanib have also failed to prove their effectiveness in the treatment of melanoma [91,92].

For c-Met or its HGF ligand, the preclinical study has shown promising results, but so far only partially confirmed in clinical trials [93,94,95]. One such inhibitor, crizotinib, was shown to be potent in preventing metastatic UM development [94]. Another kinase inhibitor, cabozantinib, showed potential clinical benefit in randomized discontinuation studies including 23 patients with metastatic UM [96]. However, these have not been confirmed in phase II randomized trials comparing the benefits of cabozantinib versus chemotherapy [97]. Another selective c-Met inhibitor, tivantinib, also showed promising results in a phase I study in patients with advanced solid tumors, including 19 with melanoma patients; Another phase I study was carried out with tepotinib in 149 patients with advanced solid tumors [98,99].

Despite many promising research results, there is still no standard of care therapy for metastatic UM with satisfactory effectiveness. More research is needed to develop new substances, better select patients for whom known treatments may be particularly effective and develop combination therapies that combine the advantages of various anti-cancer effects. It is worth mentioning tebentafusp, a bispecific fusion protein designed to target gp100 (a melanoma-associated antigen), which redirects T cells to kill tumor cells [100]. gp100 is expressed strongly in melanomas and weakly in normal melanocytes [100]. Initial clinical trial results are highly promising, even though it is restricted for patients with a particular HLA allele so far. Moreover, the technology used in the production of tebentafusp could allow any other protein to be used as a target, potentially opening the way for the production of further drugs [101].

## 14. Conclusions

UM is a relatively rare but deadly cancer. Researchers around the world continue their efforts to better understand its biology, which should translate into more effective treatment, especially of metastatic disease. The IHC on which we focused this article remains the gold standard in tissue testing. This is due to its wide availability and proven methodology. However, there are other new research methods that are also contributing to advances in the treatment of UM.

It is worth looking at the research on long non-coding RNA, which can also serve as a potential prognostic factor [102]. The use of Multi-Platform OMICS Analysis also seems prospective [103]. Single-cell analysis, single-cell RNA sequencing, and molecular profiling are also highly promising and interesting methods [104,105,106]. The development of science and wider access to new technologies enable a better understanding of the mechanisms leading to the development of all diseases, especially cancer. However, do not forget about older, proven methods such as IHC, as usually all types of tests complement each other perfectly, thus guaranteeing the best results.

## Figures and Tables

**Table 1:** Summary of all markers whose expression is related to different prognostic factors in uveal melanoma.

Marker	Protein Function	Study Group Size	Conclusion
ABCB5 (ATP-binding cassette sub-family B member 5) [17]	Human transmembrane P-glycoprotein, plays a role in transmembrane transport (including chemotherapeutic drugs).	55	High expression correlates with presence of metastasis.
ADAM10 (A disintegrin and metalloproteinase domain-containing protein 10) [18]	Transmembrane protein, controls membrane fusion and cell-cell and cell-matrix interactions.	52	High expression correlates with presence of metastasis.
Adiponectin (GBP-28, apM1, AdipoQ and Acrp30) [19]	Protein hormone involved in regulating glucose levels, fatty acid breakdown, plays a role in limiting cell proliferation and reducing inflammation.	34	Low expression correlates with extrascleral extension, more frequent chromosome 3 loss and presence of metastasis.
AIF (Apoptosis inducing factor) [20]	Ubiquitous protein, plays a proapoptotic function in the nucleus and redox activity in mitochondria.	54	High expression correlates with reduced survival time.
ATM (Ataxia-telangiectasia mutated protein) [21]	Predominantly nuclear protein, an activator of DNA damage response.	69	Loss of expression correlates with larger tumor diameter, tumor thickness, presence of epithelioid cells, reduced disease-free survival time.
ATM (Ataxia-telangiectasia mutated protein) [22]	Predominantly nuclear protein, an activator of DNA damage response.	69	Loss of expression correlates with reduced disease-free survival time.
Beclin [23]	Autophagy related protein, plays a central role in the autophagic process as a major member of the macro-autophagic phase.	85	High expression correlates with less frequent presence of metastasis and longer disease-free survival time.
BNIP3 (BCL2 19 kD protein-interacting protein 3) [24]	Cytoplasm protein, regulates cell death, autophagy, and cytoprotection.	47	High expression correlates with deeper scleral invasion, increased pigmentation and reduced overall survival time.
BTNL9 (Butyrophilin-like protein 9) [25]	Modulator of the T cell response.	62	High expression correlates with longer overall survival time.
CCR7 (C-C Motif Chemokine Receptor 7) [26]	Receptor mainly expressed in lymphoid cells, mediate cell migration of naıve lymphocytes and mature dendritic cells to secondary lymphoid organs and regulate the transport of cancer cells through the extracellular matrix.	49	High expression correlates with higher tumor thickness, presence of epithelioid cells, lymphocytic infiltration, presence of necrosis and reduced overall survival time.
CCR7 (C-C Motif Chemokine Receptor 7) [27]	Receptor mainly expressed in lymphoid cells, mediate cell migration of naıve lymphocytes and mature dendritic cells to secondary lymphoid organs and regulate the transport of cancer cells through the extracellular matrix.	70	High expression correlates with presence of metastasis.
CD147 (Cluster of differentiation 147, Basigin (BSG), extracellular matrix metalloproteinase inducer (EMMPRIN)) [28]	Member of the immunoglobulin superfamily, plays a role in intercellular recognition, various immunologic phenomena, differentiation, and development.	49	High expression in the nonmetastatic sub-group correlates with larger tumor diameter and TNM stage. In the metastatic sub-group, the presence of nested CD147 positive cells correlates with ciliary body involvement.
CEACAM (Carcinoembryonic antigen cell adhesion molecule-1) [29]	Transmembrane glycoprotein, plays a role in the intercellular interactions, regulation of cell growth, angiogenesis, immune modulation, and hepatic insulin clearance.	79	High expression correlates with presence of epithelioid cells and network extracellular matrix pattern.
c-Met (Tyrosine-protein kinase Met or hepatocyte growth factor receptor (HGFR)) [30]	Transmembrane RTK receptor, plays a role in embryonic development, organogenesis and wound healing, angiogenesis, and metastasis formation.	60	High expression correlates with presence of metastasis and reduced overall survival time.
c-Met (Tyrosine-protein kinase Met or hepatocyte growth factor receptor (HGFR)) [31]	Transmembrane RTK receptor, plays a role in embryonic development, organogenesis and wound healing, angiogenesis, and metastasis formation.	132	High expression correlates with melanoma-specific mortality.
C-NFκB proteins (Canonical nuclear factor-κB proteins (p65 and p50)) [32]	Nuclear protein, coordinator of innate immunity and inflammation.	75	High expression of p65 and p50 correlates with presence of metastasis and reduced survival time.
COX-2 (Cyclooxygenase-2) [33]	Enzyme, which catalyze the prostanoid synthesis reaction.	32	High expression correlates with metastatic death.
COX-2 (Cyclooxygenase-2) [34]	Enzyme, which catalyze the prostanoid synthesis reaction.	43	High expression correlates with presence of metastasis.
c-REL [35]	Member of the nuclear factor κB (NF-κB) transcription factor family and an emerging regulator of tumorigenesis.	75	High expression correlates with tumor thickness, presence of epithelioid cells, presence of metastasis and reduced overall survival time.
Cripto-1 (Teratocarcinoma-derived growth factor-1) [36]	An oncogenic growth factor involving tumorigenesis and cancer cell proliferation and survival.	36	High expression correlates with extrascleral extension and presence of metastasis.
CXCR4 (C-X-C motif chemokine receptor 4) [37]	Alpha-chemokine receptor specific for stromal-derived-factor-1, a molecule endowed with potent chemotactic activity for lymphocytes.	44	High expression correlates with presence of epithelioid cells.
CyclinD1 [38]	Predominantly nuclear protein, regulator of cell cycle.	66	High expression correlates with extrascleral extension and presence of epithelioid cells.
CyclinD1 [39]	Predominantly nuclear protein, regulator of cell cycle.	96	High expression correlates with extrascleral extension, presence of the mixed or epithelioid cells the tumor cell MIB-1 positivity and presence of metastasis.
EGFR (Epidermal growth factor receptor) [40]	Transmembrane protein, plays a role in epithelial tissue development and homeostasis.	40	High expression correlates with higher mitotic activity.
EGFR (Epidermal growth factor receptor) [41]	Transmembrane protein, plays a role in epithelial tissue development and homeostasis.	22	High expression correlates with metastatic death.
EMAP-II (Endothelial monocyte-activating polypeptide II) [42]	Proinflammatory cytokine and chemoattractant of macrophages, expressed on the cell surface.	25	High expression correlates with macrophage infiltration.
EphA1 (Eph-A1 receptor, erythropoietin-producing human hepatocellular receptor A1) [43]	RTK receptor, plays a role in the regulation of a processes critical to embryonic development including axon guidance, formation of tissue boundaries, cell migration, segmentation, proliferation, and angiogenesis.	94	High expression correlates with smaller tumor diameter, less frequently occurring extrascleral extension, lower mitotic activity, and presence of vitreous hemorrhage.
EphA5 (Eph-A5 receptor, erythropoietin-producing human hepatocellular receptor A5) [43]	RTK receptor, plays a role in the regulation of a processes critical to embryonic development including axon guidance, formation of tissue boundaries, cell migration, segmentation, proliferation, and angiogenesis.	94	High expression correlates with less frequent chromosome 3 loss, more frequent occurrence of vitreous hemorrhage, absence of distant metastases and longer overall survival time.
HER3 (Human epidermal growth factor receptor 3 or receptor tyrosine-protein kinase erbB-3) [44]	Transmembrane RTK receptor, implicated in growth, proliferation, chemotherapeutic resistance, and the promotion of invasion and metastasis.	128	High nuclear expression correlates with longer overall survival time.
HERC2 (HECT and RLD Domain Containing E3 Ubiquitin Protein Ligase 2) [45]	Predominantly nuclear and cytoplasm protein, plays a role in DNA repair regulation, pigmentation, and neurological disorders.	52	High expression correlates with ciliary body involvement, presence of epithelioid cells and increased pigmentation.
Hsp90 (Heat shock protein 90) [46]	Cytoplasmic protein, plays a role in folding, intracellular transport, maintenance, and degradation of proteins, and facilitating cell signaling.	44	High expression correlates with larger tumor diameter.
ICAM-1 (Intercellular cell adhesion molecule-1) [47]	Adhesion molecule, ligand for leukocyte function-associated antigen-1, involved in the process of inflammation, the circulation of blood cells, and in the immune surveillance of the host.	90	Loss of expression correlates with presence of metastasis.
IGF-1R (Insulin-like growth factor 1 receptor) [31]	Transmembrane receptor, implicated in insulin signaling, plays a role in several cancer development.	132	High expression correlates with melanoma-specific mortality.
IGF-1R (Insulin-like growth factor 1 receptor) [48]	Transmembrane receptor, implicated in insulin signaling, plays a role in several cancer development.	36	High expression correlates with death of disease.
JARID1B (Jumonji AT-rich interactive domain 1B) [49]	Demethylase enzyme, induce demethylation of tri- and di-methylated lysines in the 4 position of histone 3.	121	High expression correlates with reduced survival time.
LOX (Lysyl oxidase) [50]	Extracellular enzyme, plays a role in embryonic development, wound healing and adult tissue remodeling.	33	High expression correlates with presence of epithelioid cells and reduced metastasis-free survival time.
MCAM (Melanoma cell adhesion molecule, MUC18, Mel-Cam, CD146) [51]	Adhesion molecule, plays a role in intracellular signaling cascades.	35	High expression correlates with death of disease.
MMP-2 and MMP-9 (Matrix metalloproteinase-2 and -9) [28]	Secreted and membrane-associated neutral endopeptidase, plays a role in degrading extracellular matrix proteins, cell proliferation, migration, differentiation, angiogenesis, apoptosis, and host defense.	26	High expression correlates with presence of metastasis and reduced survival time.
MMP-9 (Matrix metalloproteinase 9) [52]	Secreted and membrane-associated neutral endopeptidase, plays a role in degrading extracellular matrix proteins, cell proliferation, migration, differentiation, angiogenesis, apoptosis, and host defense.	100	High expression correlates with presence of loop and/or network patterns, lymphocytic infiltration, presence of necrosis and presence of metastasis.
Nbs1 (Nibrin, NBN) [53]	Intracellular protein, plays a role in the repair of double strand breaks and telomere maintenance.	49	High expression correlates with reduced survival time.
NC-NFκB proteins (p52, RelB, and co-expression of p52/RelB) [54]	Nuclear protein, plays a role in promoting cancer proliferation and progression.	75	High expression correlates with reduced metastasis-free survival time and reduced overall survival time.
NEMO/IKKγ (Factor κB essential modulator, inhibitor of nuclear factor kappa B kinase subunit gamma) [55]	Protein essential for the activation of transcription factor NFκB, which regulates the cellular responses to inflammation, immunity, and cell survival.	75	Low expression correlates with presence of epithelioid cells, higher mitotic activity, presence of vascular loop, neovascularization and reduced overall survival time.
Nestin (Neural stem cell protein) [56]	Cytoplasm and membrane-bound protein, member of the intermediate filament (IF) class VI protein family	167	High expression correlates with presence of epithelioid cells, more frequent chromosome 3 loss and chromosome 8q gain, higher mitotic activity, presence of vascular loop and reduced survival time.
nm23 (Nucleoside diphosphate kinase A) [57]	Cytoplasm protein, product of metastasis suppressor gene (*NM23*).	33	Low expression correlates with larger tumor diameter and deeper scleral invasion.
nm23-H1 (Nucleoside diphosphate kinase A) [58]	Cytoplasm protein, product of metastasis suppressor gene (*NM23*).	32	The increased immunostaining intensity correlates with longer survival time.
p16 (Cyclin-dependent kinase inhibitor 2A, CDKN2A, multiple tumor suppressor 1) [59]	Predominantly nuclear protein, slowing the progression of the cell cycle from G1 phase to the S phase.	41	Low expression correlates with mixed cell type.
p53 [39]	Cell-cycle regulatory protein.	96	High expression correlates with presence of metastasis.
PARP (Poly (ADP-ribose) polymerase) [60]	Nuclear protein, which participate in the DNA repair processes.	91	High expression correlates with larger tumor diameter, higher histopathological grade, more frequent chromosome 3 loss, reduced overall survival time and disease-free survival time.
PCNA (Proliferating cell nuclear antigen, ATLD2) [61]	The DNA polymerase auxiliary protein involved in the control of DNA replication.	212	High expression correlates with presence of metastasis.
PD-1 (Programmed cell death receptor-1) [62]	Membrane-bound receptor, plays a role in regulation immune system’s response to the cells of the human body.	71	High expression correlates with ciliary body involvement, presence of epithelioid cells, macrophage infiltration, absence of BAP-1 staining and presence of metastasis.
PD-1 (Programmed cell death receptor-1) [63]	Membrane-bound receptor, plays a role in regulation immune system’s response to the cells of the human body.	82	High expression correlates with larger tumor diameter and reduced survival time.
PD-L1 (Programmed death-ligand 1) [62]	Ligand for PD-1 (programmed cell death receptor-1), plays a role in regulation immune system’s response to the cells of the human body.	71	High expression correlates with macrophage infiltration, higher AJCC prognostic stage group and presence of metastasis.
PERP (p53 apoptosis effector related to PMP-22) [64]	Cytoplasm protein, plays a role in inducing cell death.	16	Low expression correlates with more frequent chromosome 3 loss.
Phospho-Akt [65]	Cytoplasmic protein, plays a role in phosphorylation and inactivation of several proteins involved in apoptosis.	34	High expression correlates with presence of metastasis.
PLK-1 (Polo-like kinase-1) [66]	Regulator of mitotic entry and cytokinesis.	158	Low expression correlates with higher clinical tumor stage, higher AJCC prognostic stage group and reduced overall survival time.
P-protein (Pink-eyeddilution protein) [45]	Membrane protein, plays a role in melanin synthesis in melanocytes and retinal pigment epithelium.	52	High expression correlates with ciliary body involvement, presence of epithelioid cells, increased pigmentation, and advanced clinical tumor staging. High cytoplasmic expression correlates with presence of metastasis.
PRDX3 (Thioredoxin-dependent peroxidase reductase) [67]	Cytoplasmic protein, involved in redox regulation of the cell and protects radical-sensitive enzymes from oxidative damage.	92	High expression correlates with presence of metastasis and reduced survival time.
RKIP (Raf Kinase Inhibitor Protein) [68]	Regulator of proliferative pathways within the cell.	44	Low expression correlates with presence of metastasis.
SPANX-C (SPANX family member C; Sperm protein associated with the nucleus on the X chromosome C) [69]	Cytoplasmic protein, expressed in highly metastatic cell lines.	55	High expression correlates with presence of metastasis.
SSR (Somatostatin receptor, SSTR) [70]	The binding of somatostatin to its membrane receptor starts a signaling pathway that leads to arrest of cell growth or apoptosis.	25	High expression correlates with longer survival time.
Syntenin (Syndecan binding protein syntenin-1, melanoma differentiation-associated gene 9, mda-9) [71]	Predominantly cytoplasm protein, plays a role in clustering of membrane receptors, intracellular trafficking, Sox4 activation, and signal transduction.	29	High expression correlates with risk of metastasis recurrence.
TIMP-1 and TIMP-2 (Tissue inhibitor of metalloproteinase-1 and -2) [28]	Metalloproteinase inhibitors.	26	High expression correlates with longer survival time.
VEGF-A (Vascular endothelial growth factor-A) [52]	Acts specifically on endothelial cells, mediates increased vascular permeability, induces angiogenesis, vasculogenesis and endothelial cell growth, promotes cell migration, and inhibits apoptosis.	100	High expression correlates with presence of vascular loos and/or network patterns, lymphocytic infiltration, necrosis, and presence of metastasis.

**Table 2:** Summary of markers whose expression is related to tumor size.

Marker	Study Group Size	Conclusion
EphA1 (Eph-A1 receptor, erythropoietin-producing human hepatocellular receptor A1) [43]	94	High expression correlates with smaller tumor diameter.
nm23 (Nucleoside diphosphate kinase A) [57]	33	Low expression correlates with larger tumor diameter.
CD147 (Cluster of differentiation 147, Basigin (BSG), extracellular matrix metalloproteinase inducer (EMMPRIN)) [28]	49	High expression in the nonmetastatic sub-group correlates with larger tumor diameter.
Hsp90 (Heat shock protein 90) [46]	44	High expression correlates with larger tumor diameter.
PARP (Poly (ADP-ribose) polymerase) [60]	91	High expression correlates with larger tumor diameter.
PD-1 (Programmed cell death receptor-1) [63]	82	High expression correlates with larger tumor diameter.
ATM (Ataxia-telangiectasia mutated protein) [21]	69	Loss correlates with larger tumor diameter and tumor thickness.
CCR7 (C-C Motif Chemokine Receptor 7) [26]	49	High expression correlates with tumor thickness.
c-REL [35]	75	High expression correlates with tumor thickness.

**Table 3:** Summary of markers whose expression is related to ciliary body involvement.

Marker	Study Group Size	Conclusion
CD147 (Cluster of differentiation 147, Basigin (BSG), extracellular matrix metalloproteinase inducer (EMMPRIN)) [28]	49	In the metastatic sub-group, the presence of nested CD147 positive cells correlates with ciliary body involvement.
HERC2 (HECT and RLD Domain Containing E3 Ubiquitin Protein Ligase 2) [45]	52	High expression correlates with ciliary body involvement.
PD-1 (Programmed cell death receptor-1) [62]	71	High expression correlates with ciliary body involvement.
P-protein (Pink-eyed dilution protein) [45]	52	High expression correlates with ciliary body involvement.

**Table 4:** Summary of markers whose expression is related to scleral and extrascleral infiltration.

Marker	Study Group Size	Conclusion
BNIP3 (BCL2 19 kD protein-interacting protein 3) [24]	47	High expression correlates with deeper scleral invasion.
nm23 (Nucleoside diphosphate kinase A) [57]	33	Low expression correlates with deeper scleral invasion.
Adiponectin (GBP-28, apM1, AdipoQ and Acrp30) [19]	34	Low expression correlates with extrascleral extension.
Cripto-1 (Teratocarcinoma-derived growth factor-1) [36]	36	High expression correlates with extrascleral extension.
CyclinD1 [38]	66	High expression correlates with extrascleral extension.
CyclinD1 [39]	96	High expression correlates with extrascleral extension.
EphA1 (Eph-A1 receptor, erythropoietin-producing human hepatocellular receptor A1) [43]	94	High expression correlates with less frequently occurring extrascleral extension.

**Table 5:** Summary of markers whose expression is related to presence of various cell types.

Marker	Study Group Size	Conclusion
ATM (Ataxia-telangiectasia mutated protein) [21]	69	Low expression correlates with presence of epithelioid cells.
CCR7 (C-C Motif Chemokine Receptor 7) [26]	49	High expression correlates with presence of epithelioid cells.
CEACAM (Carcinoembryonic antigen cell adhesion molecule-1) [29]	79	High expression correlates with presence of epithelioid cells.
c-REL [35]	75	High expression correlates with presence of epithelioid cells.
CXCR4 (C-X-C motif chemokine receptor 4) [37]	44	High expression correlates with presence of epithelioid cells.
CyclinD1 [38]	66	High expression correlates with presence of epithelioid cells.
HERC2 (HECT and RLD Domain Containing E3 Ubiquitin Protein Ligase 2) [45]	52	High expression correlates with presence of epithelioid cells.
LOX (Lysyl oxidase) [50]	33	High expression correlates with presence of epithelioid cells.
NEMO/IKKγ (Nuclear factor κB essential modulator, inhibitor of nuclear factor kappa B kinase subunit gamma) [55]	75	Low expression correlates with presence of epithelioid cells.
Nestin (Neural stem cell protein) [56]	167	High expression correlates with presence of epithelioid cells.
PD-1 (Programmed cell death receptor-1) [62]	71	High expression correlates with presence of epithelioid cells.
P-protein (Pink-eyed dilution protein) [45]	52	High expression correlates with presence of epithelioid cells.
CyclinD1 [39]	96	High expression correlates with presence of mixed or epithelioid cells.
p16 (Cyclin-dependent kinase inhibitor 2A, CDKN2A, multiple tumor suppressor 1) [59]	41	Low expression correlates with mixed cell type.
PARP (Poly (ADP-ribose) polymerase) [60]	91	High expression correlates with higher histopathological grade.

**Table 6:** Summary of markers whose expression is related to chromosome status.

Marker	Study Group Size	Conclusion
EphA5 (Eph-A5 receptor, erythropoietin-producing human hepatocellular receptor A5) [43]	94	High expression correlates with less frequent chromosome 3 loss.
Nestin (Neural stem cell protein) [56]	167	High expression correlates with more frequent chromosome 3 loss and chromosome 8q gain.
PARP (Poly (ADP-ribose) polymerase) [60]	91	High expression correlates with more frequent chromosome 3 loss.
Adiponectin (GBP-28, apM1, AdipoQ and Acrp30) [19]	34	Low expression correlates with more frequent chromosome 3 loss.
PERP (p53 apoptosis effector related to PMP-22) [64]	16	Low expression correlates with more frequent chromosome 3 loss.

**Table 7:** Summary of markers whose expression is related to mitotic count.

Marker	Study Group Size	Conclusion
EGFR (Epidermal growth factor receptor) [40]	40	High expression correlates with higher mitotic count.
EphA1 (Eph-A1 receptor, erythropoietin-producing human hepatocellular receptor A5) [43]	94	High expression correlates with lower mitotic count.
NEMO/IKKγ (Nuclear factor κB essential modulator, inhibitor of nuclear factor kappa B kinase subunit gamma) [55]	75	Low expression correlates with higher mitotic count.
Nestin (Neural stem cell protein) [56]	167	High expression correlates with higher mitotic count.

**Table 8:** Summary of markers whose expression is related to extravascular matrix loops and networks.

Marker	Study Group Size	Conclusion
CEACAM (Carcinoembryonic antigen cell adhesion molecule-1) [29]	79	High expression correlates with network extracellular matrix pattern.
MMP-9 (Matrix metalloproteinase-9) [52]	100	High expression correlates with presence of vascular loops and network patterns.
VEGF-A (Vascular endothelial growth factor-A) [52]	100	High expression correlates with presence of vascular loops and network patterns.
NEMO/IKKγ (Nuclear factor κB essential modulator, inhibitor of nuclear factor kappa B kinase subunit gamma) [55]	75	Low expression correlates with presence of vascular loops.
Nestin (Neural stem cell protein) [56]	167	High expression correlates with presence of vascular loops.

**Table 9:** Summary of markers whose expression is related to TIMs and TILs infiltration.

Marker	Study Group Size	Conclusion
EMAP-II (Endothelial monocyte-activating polypeptide II) [42]	25	High expression correlates with macrophage infiltration.
PD-1 (Programmed cell death receptor-1) [62]	71	High expression correlates with macrophage infiltration.
PD-L1 (Programmed death-ligand 1) [62]	71	High expression correlates with macrophage infiltration.
CCR7 (C-C Motif Chemokine Receptor 7) [26]	49	High expression correlates with lymphocytic infiltration.
MMP-9 (Matrix metalloproteinase-9) [52]	100	High expression correlates with lymphocytic infiltration.
VEGF-A (Vascular endothelial growth factor-A) [52]	100	High expression correlates with lymphocytic infiltration.

**Table 10:** Summary of markers whose expression is related to other prognostic factors.

Marker	Study Group Size	Conclusion
BNIP3 (BCL2 19 kD protein-interacting protein 3) [24]	47	High expression correlates with increased pigmentation.
HERC2 (HECT and RLD Domain Containing E3 Ubiquitin Protein Ligase 2) [45]	52	High expression correlates with increased pigmentation.
P-protein (Pink-eyed dilution protein) [45]	52	High expression correlates with increased pigmentation and advanced clinical tumor staging.
PD-L1 (Programmed death-ligand 1) [63]	71	High expression correlates with higher AJCC prognostic stage group.
PLK-1 (Polo-like kinase-1) [66]	158	Low expression correlates with higher AJCC prognostic stage group.
CD147 (Cluster of differentiation 147, Basigin (BSG), extracellular matrix metalloproteinase inducer (EMMPRIN)) [28]	49	High expression in the nonmetastatic sub-group correlates with TNM stage.
CCR7 (C-C Motif Chemokine Receptor 7) [26]	49	High expression correlates with necrosis.
MMP-9 (Matrix metalloproteinase-9) [52]	100	High expression correlates with degree of necrosis.
VEGF-A (Vascular endothelial growth factor-A) [52]	100	High expression correlates with degree of necrosis.
EphA1 (Eph-A1 receptor, erythropoietin-producing human hepatocellular receptor A1) [43]	94	High expression correlates with more frequent presence of hemorrhage in the vitreous chamber.
EphA5 (Eph-A5 receptor, erythropoietin-producing human hepatocellular receptor A5) [43]	94	High expression correlates with more frequent presence of hemorrhage in the vitreous chamber.
NEMO/IKKγ (Nuclear factor κB essential modulator, inhibitor of nuclear factor kappa B kinase subunit gamma) [55]	75	Low expression correlates with neovascularization.
CyclinD1 [39]	96	High expression correlates with the tumor cell MIB-1 positivity.
PD-1(Programmed cell death receptor-1) [62]	71	High expression correlates with absence of BAP-1 staining.

**Table 11:** Summary of markers whose expression is related to metastasis presence.

Marker	Study Group Size	Conclusion
ABCB5 (ATP-binding cassette sub-family B member 5) [17]	55	High expression correlates with presence of metastasis.
ADAM10 (A disintegrin and metalloproteinase domain-containing protein 10) [18]	52	High expression correlates with presence of metastasis.
Adiponectin (GBP-28, apM1, AdipoQ and Acrp30) [19]	34	Low expression correlates with presence of metastasis.
Beclin [23]	85	High expression correlates with less frequent metastasis.
CCR7 (C-C Motif Chemokine Receptor 7) [27]	70	High expression correlates with presence of metastasis.
c-Met (Tyrosine-protein kinase Met or hepatocyte growth factor receptor (HGFR)) [30]	60	High expression correlates with presence of metastasis.
C-NFκB proteins (Canonical nuclear factor-κB proteins (p65 and p50)) [32]	75	High expression correlates with presence of metastasis.
COX-2 (Cyclooxygenase-2) [33]	32	High expression correlates with metastatic death.
COX-2 (Cyclooxygenase-2) [34]	43	High expression correlates with presence of metastasis.
c-REL [35]	75	High expression correlates with presence of metastasis.
Cripto-1 (Teratocarcinoma-derived growth factor-1) [36]	36	High expression correlates with presence of metastasis.
CyclinD1 [39]	96	High expression correlates with presence of metastasis.
EGFR (Epidermal growth factor receptor) [41]	22	High expression correlates with metastatic death.
EphA5 (Eph-A5 receptor, erythropoietin-producing human hepatocellular receptor A5) [43]	94	High expression correlates with less frequent metastasis.
ICAM-1 (Intercellular cell adhesionmolecule-1) [47]	90	Loss of expression correlates with presence of metastasis.
IGF-1R (Insulin-like growth factor 1 receptor) [48]	36	High expression correlates with metastatic death.
LOX (Lysyl oxidase) [50]	33	High expression correlates with reduced metastasis-free survival time.
MCAM (Melanoma cell adhesion molecule, MUC18, Mel-Cam, CD146) [51]	35	High expression correlates with metastatic death.
MMP-2 and MMP-9 (Matrix metalloproteinase-2 and -9) [28]	26	High expression correlates with presence of metastasis.
MMP-9 (Matrix metalloproteinase-9) [52]	100	High expression correlates with presence of metastasis.
NC-NFκB proteins (p52, RelB, and co-expression of p52/RelB) [54]	75	High expression correlates with reduced metastasis-free survival time.
p53 [39]	96	High expression correlates with presence of metastasis.
PCNA (Proliferating cell nuclear antigen, ATLD2) [61]	212	High expression correlates with presence of metastasis.
PD-1 (Programmed cell death receptor-1) [62]	71	High expression correlates with presence of metastasis.
PD-L1 (Programmed death-ligand 1) [62]	71	High expression correlates with presence of metastasis.
phospho-Akt [65]	34	High expression correlates with presence of metastasis.
P-protein (Pink-eyed dilution protein) [45]	52	High cytoplasmic expression correlates with presence of metastasis.
PRDX3 (Thioredoxin-dependent peroxidase reductase) [67]	92	High expression correlates with presence of metastasis.
RKIP (Raf Kinase Inhibitor Protein) [68]	44	Low expression correlates with presence of metastasis.
SPANX-C (SPANX family member C; Sperm protein associated with the nucleus on the X chromosome C) [69]	55	High expression correlates with presence of metastasis.
Syntenin (Syndecan binding protein syntenin-1, melanoma differentiation-associated gene 9, mda-9) [71]	29	High expression correlates with risk of metastasis recurrence.
VEGF-A (Vascular endothelial growth factor-A) [52]	100	High expression correlates with presence of metastasis.

**Table 12:** Summary of markers whose expression is related to survival times.

Marker	Study Group Size	Conclusion
AIF (Apoptosis inducing factor) [20]	54	High expression correlates with reduced survival time.
ATM (Ataxia-telangiectasia mutated protein) [21]	69	Loss of expression correlates with reduced disease-free survival time.
ATM (Ataxia-telangiectasia mutated protein) [22]	69	Loss of expression correlates with reduced disease-free survival time.
Beclin [23]	85	High expression correlates with longer disease-free survival time.
BNIP3 (BCL2 19 kD protein-interacting protein 3) [24]	47	High expression correlates with reduced overall survival time.
BTNL9 (Butyrophilin-like protein 9) [25]	62	High expression correlates with longer overall survival time.
CCR7 (C-C Motif Chemokine Receptor 7) [26]	49	High expression correlates with reduced overall survival time.
c-Met (Tyrosine-protein kinase Met or hepatocyte growth factor receptor (HGFR)) [30]	60	High expression correlates with reduced overall survival time.
c-Met (Tyrosine-protein kinase Met or hepatocyte growth factor receptor (HGFR)) [31]	132	High expression correlates with melanoma-specific mortality.
C-NFκB proteins (Canonical nuclear factor-κB proteins (p65 and p50)) [32]	75	High expression correlates with reduced survival time.
c REL [35]	75	High expression correlates with reduced overall survival time.
EphA5 (Eph-A5 receptor, erythropoietin-producing human hepatocellular receptor A5) [43]	94	High expression correlates with longer overall survival time.
HER3 (Human epidermal growth factor receptor 3 or receptor tyrosine-protein kinase erbB-3) [44]	128	High nuclear expression correlates with longer overall survival time.
IGF-1R (Insulin-like growth factor 1 receptor) [31]	132	High expression correlates with melanoma-specific mortality.
JARID1B (Jumonji AT-rich interactive domain 1B) [49]	121	High expression correlates with reduced survival time.
Legumain (Asparagine endopeptidase (AEP)) [76]	82	High expression correlates with reduced survival time.
MMP-2 and MMP-9 (Matrix metalloproteinase-2 and -9) [28]	26	High expression correlates with reduced survival time.
Nbs1 (Nibrin, NBN) [53]	49	High expression correlates with reduced survival time.
NC-NFκB proteins (p52, RelB, and co-expression of p52/RelB) [54]	75	High expression correlates with reduced overall survival time.
NEMO/IKKγ (Nuclear factor κB essential modulator, inhibitor of nuclear factor kappa B kinase subunit gamma) [55]	75	Low expression correlates with reduced overall survival time.
Nestin (Neural stem cell protein) [56]	167	High expression correlates with reduced survival time.
nm23-H1 (Nucleoside diphosphate kinase A) [58]	32	The increased immunostaining intensity correlates with longer survival time.
PARP (Poly (ADP-ribose) polymerase) [60]	91	High expression correlates with reduced overall survival time and disease-free survival time.
PD-1 (Programmed cell death receptor-1) [63]	82	High expression correlates with reduced survival time.
PLK-1 (Polo-like kinase-1) [66]	158	Low expression correlates with reduced overall survival time.
PRDX3 (Thioredoxin-dependent peroxidase reductase) [67]	92	High expression correlates with reduced survival time.
SSR (Somatostatin receptor, SSTR) [70]	25	High expression correlates with longer survival time.
TIMP-1 and TIMP-2 (Tissue inhibitor of metalloproteinase-1 and -2) [28]	26	High expression correlates with longer survival time.
